# Hematopoietic Stem Cell Function in a Murine Model of Sickle Cell Disease

**DOI:** 10.1155/2012/387385

**Published:** 2012-06-04

**Authors:** Elisabeth H. Javazon, Mohamed Radhi, Bagirath Gangadharan, Jennifer Perry, David R. Archer

**Affiliations:** ^1^Department of Biology, Morehouse College, 830 Westview Drive Southwest, Atlanta, GA 30314-3773, USA; ^2^Department of Pediatrics, UI Hospitals and Clinics, University of Iowa, 2633 Carver Pavilion, 200 Hawkins Drive, Iowa City, IA 52242, USA; ^3^Aflac Cancer and Blood Disorders Center, Emory University and Children's Healthcare of Atlanta, 2015 Uppergate Drive, Atlanta, GA 30322, USA

## Abstract

Previous studies have shown that the sickle environment is highly enriched for reactive oxygen species (ROS). We examined the oxidative effects of sickle cell disease on hematopoietic stem cell function in a sickle mouse model. *In vitro* colony-forming assays showed a significant decrease in progenitor colony formation derived from sickle compared to control bone marrow (BM). Sickle BM possessed a significant decrease in the KSL (c-kit^+^, Sca-^1+^, Lineage^−^) progenitor population, and cell cycle analysis showed that there were fewer KSL cells in the G_0_ phase of the cell cycle compared to controls. We found a significant increase in both lipid peroxidation and ROS in sickle-derived KSL cells. *In vivo* analysis demonstrated that normal bone marrow cells engraft with increased frequency into sickle mice compared to control mice. Hematopoietic progenitor cells derived from sickle mice, however, demonstrated significant impairment in engraftment potential. We observed partial restoration of engraftment by n-acetyl cysteine (NAC) treatment of KSL cells prior to transplantation. Increased intracellular ROS and lipid peroxidation combined with improvement in engraftment following NAC treatment suggests that an altered redox environment in sickle mice affects hematopoietic progenitor and stem cell function.

## 1. Introduction

Sickle cell disease (SCD) is one of the most common inherited hemoglobinopathies in the world. In the United States, approximately 1 in 600 African Americans have been diagnosed with SCD [1]. SCD is an autosomal recessive genetic disorder caused by a substitution of glutamic acid by valine in the beta subunit of the hemoglobin gene. This substitution results in the production of abnormal hemoglobin (HbS). Deoxygenated HbS polymerizes, resulting in intravascular hemolysis of the red blood cell and release of hemoglobin and other compounds into the plasma [2]. Repeating cycles of polymerization and hemolysis lead to vaso-occlusion and ischemia-reperfusion injury. Inherent in these processes are inflammatory responses and oxidant stress which result in pathological outcomes such as acute chest syndrome, pulmonary hypertension, and stroke in patients with SCD [3].

Oxidative stress is a result of increased production of reactive oxygen species (ROS) combined with decreased production or availability of antioxidants. Cellular metabolism of oxygen can lead to the production of ROS such as superoxide anion radicals, hydrogen peroxide, and hydroxyl radicals. ROS can impair the proper function of DNA, lipids, proteins, and carbohydrates [4]. An imbalance in the amount of pro-oxidants and antioxidants leads to an environment of oxidative stress, cell dysfunction, or cell death. Antioxidants such as nitric oxide (NO), superoxide dismutase (SOD), and reduced glutathione (GSH) function to neutralize excess ROS.

There is increasing evidence that oxidative stress and ROS play a pivotal role in the pathophysiology of numerous diseases including neurodegenerative diseases, cardiovascular diseases, cancers, and arthritis [5–10]. Oxidative stress has been linked to vascular defects leading to hypertension and atherosclerosis as well as cardiac defects leading to contractile dysfunction and dysrhythmias [11]. In addition to the ability to induce mutations in DNA, ROS play a key role in cell signaling and cell regulatory pathways and thus play a pivotal role in the development of tumors and malignancies [12–16].

In SCD, under low-oxygen conditions, HbS polymerizes leading to hemolysis and a significantly shortened lifespan [17]. Hemolysed RBCs release hemoglobin, iron, and arginase into the plasma resulting in decreased nitric oxide availability and leading to imbalanced vascular homeostasis and oxidative stress [17–19]. Sickle RBCs generate more superoxide, hydrogen peroxide, and lipid oxidation products compared to normal RBCs [20]. Increased ROS in platelets and polymorphonuclear neutrophils along with decreased glutathione levels has also been documented in patients with SCD [21]. In addition, endothelium exposed to sickled RBCs become activated, causing sickled RBCs and leukocytes to adhere to the activated endothelium, resulting in a release of cytokines and ROS [6, 22, 23]. In this study, we studied the effects of this pro-oxidant and proinflammatory environment on hematopoietic progenitor and stem cells in the Berkeley model of sickle cell disease.

## 2. Materials and Methods

### 2.1. Animals

**Figure 1:**
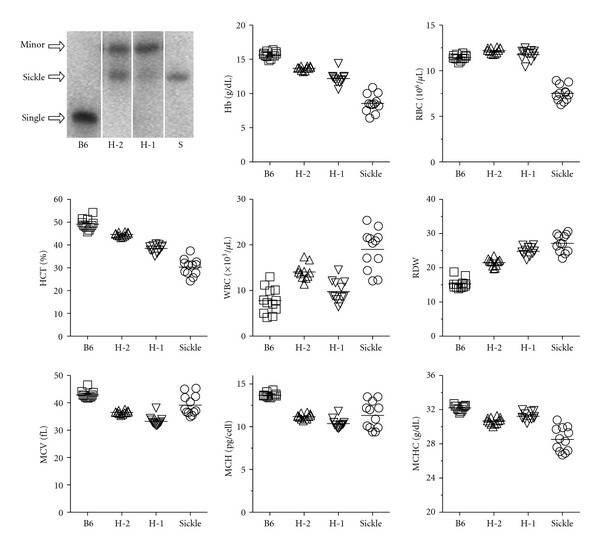
Hemoglobin and hematology profile of Berkeley sickle mice. Hemoglobin electrophoresis of RBC from C57BL/6, H-2, H-1, and homozygous sickle mice. C57BL/6 mice show a “single” hemoglobin band, whereas the H-2 and H-1 hemizygotes show beta-sickle globin resulting from two or one copy of the transgene, respectively, in combination with the minor band of diffuse beta-globins. Homozygous sickle mice only have the characteristic band of beta-sickle globin. The complete blood counts of C57BL/6 mice, H-2, H-1, and homozygous sickle mice (*n* = 10 per genotype) are also shown with the hemizygous mice having intermediate values between C57BL/6 mice and homozygous sickle mice.

Sickle mice, originally supplied by Dr. Pászty [24], express exclusively human *α*-, *β* ^sickle^, and *γ*-globin and exist on a mixed genetic background (FVB/N, 129, DBA/2, C57BL/6, and Black Swiss). Breeding and pregnant sickle mice were fed TestDiet no. 0007573 (Purina). The colony is maintained by breeding female hemizygous mice with 2 copies of the transgene to homozygous male mice. The resulting pups are hemizygous, expressing one (H-1) or two (H-2) copies of the transgene, one copy of murine beta globin, but no expression of murine alpha globin. Homozygous “sickle” mice that only express human gamma, alpha, and beta^sickle^ globins are easily distinguishable from wild type and the H-1 and H-2 hemizygotes by hemoglobin electrophoresis (Figure 1) [24, 25]. H-1 mice have ~28% sickle hemoglobin and H-2 mice have ~45%. Despite having only one copy of the sickle hemoglobin transgene, H-1 mice are hematologically more severe (Figure 1). H-2 and C57BL/6 were primarily used as control mice for the homozygous mice. All breeding and experimental procedures were performed at Emory University in accordance with the recommendations of the Institutional Animal Care and Use Committee (IACUC).

### 2.2. Flow Cytometry

Bone marrow was harvested and stained with the following fluorochrome-conjugated antibodies: CD45.1-PE, CD45.2-APC, c-kit-FITC, Sca1-PE, and Lineage-APC (B220, CD3, CD11b, GR-1, and Ter119; BD Pharmingen, San Diego, CA), and KSL cells were sorted on a FACSAria. Cells from the peripheral blood were analyzed at various time points on an LSR II flow cytometer (BD Biosciences, San Jose, CA). Propidium iodide was used to exclude dead cells. For ROS analysis, cells were incubated in 160 *μ*M Dichlorodihydrofluorescein diacetate (H_2_DCFDA) (St. Louis, MO, Sigma-Aldrich) or 5 *μ*M *N*-(fluorescein-5thiocarbamoyl)-1,2-dihexadecanoyl-*sn*-glycero-3-phosphoethanolamine, triethylammonium salt (DHPE) (Carlsbad, CA, Invitrogen) in HBSS for 15 min or 60 min, respectively, at 37°C shaking in the dark. Cells were washed and analyzed by flow cytometry. Absolute cell counts were performed using Trucount tubes (BD Biosciences).

### 2.3. HPP Assay

Twenty thousand freshly isolated bone marrow mononuclear cells were plated in methylcellulose medium (StemCell Technologies Inc., Vancouver) containing recombinant cytokines (100 ng rat steel factor, 1600U m-CSF, 75U IL-3, 5000U IL-1*α*, 30 ng/mL EGF, and 30 ng/mL FGF) to analyze the colony-forming potential of stem cells derived from the bone marrow of sickle and control mice. Cells were cultured for 10 days, and colonies were counted using an inverted microscope. 

### 2.4. Hemoglobin Analysis

Differential hemoglobin electrophoresis of peripheral blood from Berkeley sickle mice was performed to determine hetero- and homozygosity of the murine and human globin genes (Helena Titan III electrophoresis system, Helena Laboratories, Beaumont, TX) [26]. As shown in Figure 1, C57BL/6 mice express the murine beta globin “single” allele, the H-1 and H-2 hemizygotes express increasing amounts of human beta^sickle^ globin in the presence of the murine allele expressing “diffuse” beta globin, and the mice homozygous for the deletions of murine alpha and beta globins express only human beta^sickle^ globin.

### 2.5. Transplants

#### 2.5.1. The Effect of the Recipient Environment

To test the effects of transplanting into the sickle microenvironment, 1 × 10^7^ bone marrow cells from C57BL/6 mice (expressing CD45.2) were transplanted into homozygous sickle (*n* = 8) and B6.SJL-Ptprc^a^ Pepc^b^/BoyJ mice (*n* = 10; both expressing CD45.1) without ablation. While sickle and C57BL/6 share the MCH class I allele H-2K^b^, their genetic backgrounds are not identical and therefore are unlikely to share the full haplotype. We have previously shown that T-cell costimulation blockade promotes engraftment across allo-barriers in this model, and it was therefore included in this protocol. Specifically, 500 *μ*g each of hamster antimouse-CD40L (MR1; BioExpress, Lebanon, NH) and human CTLA4-immunoglobulin (generous gift from Dr. C. Larsen) was given intraperitoneally on days 0, 2, 4, and 7 relative to BMT.

#### 2.5.2. Competitive Repopulation Study

To test the repopulation capacity of individual stem cell populations, three thousand KSL cells derived from homozygous H-2 sickle, or B6.SJL-Ptprc^a^ Pepc^b^/BoyJ, were sorted using a FACSAria (BD Biosciences, San Jose, CA) and were cotransplanted with 2 × 10^6^ competitive bone marrow cells derived from C57BL/6 mice. Due to the potential differences in background, F1 recipient mice were generated by breeding H-2 sickle mice (expressing CD45.1) to C57BL/6 mice (expressing GFP under the beta globin promoter and CD45.2). These recipient mice expressed GFP+, CD45.1, and CD45.2 double positive cells (F1 mice). All recipients (*n* = 4 each group) were conditioned with two doses of 550 cGy total body irradiation on the day of BMT. To test the ability of the glutathione precursor to alleviate oxidant stress in the selected stem cell populations, groups of donor mice were also treated for four weeks with 0.163 g/L n-acetyl cysteine (NAC) in their drinking water (fresh NAC drinking water was made every two days). Mice were 12 weeks of age at the time of bone marrow harvest and transplantation.

## 3. Statistics

All statistical comparisons were performed using GraphPad Prism software utilizing one-way ANOVA with a Tukey posttest analysis unless otherwise stated (*P < 0.05; **P < 0.01; ***P < 0.001).

## 4. Results

### 4.1. Stem Cell Number and Function

**Figure 2:**
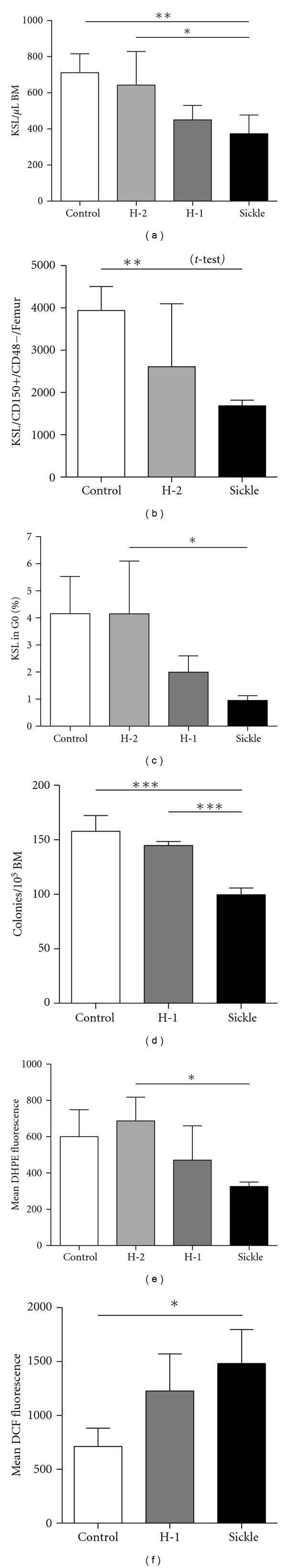
Stem cell number and oxidant state in Berkeley sickle mice. (a) Quantification of KSL progenitor cells in the bone marrow of control, hemizygous, and homozygous sickle mice showing a significant reduction in number in homozygous sickle mice. (b) Further examination of a phenotypically defined HSC population (KSL/CD150+/CD48−) also shows a reduction of HSC in sickle BM. (c) HSCs in the stem cell niche are known to be quiescent, and homozygous mice again show significant reduction in KSL cells in the G_0_ phase of the cell cycle possibly indicating the mobilization of sickle progenitor cells. (d) The *in vitro* colony-forming, high proliferative potential assay (HPP) of stem/progenitor cells further indicates a significant reduction in colony-forming cells in homozygous mice. (e) To examine the effect of oxidant stress on hematopoietic progenitors, we measured lipid peroxidation using the fluorescent indicator DHPE. A reduction in fluorescence indicates increased activity of hydroxyl radicals. (f) Further quantification of reactive oxygen species was shown in sickle mice by the increased production of DCF (all graphs are presented as mean ± SD, *P < 0.05, **P < 0.01, ***P < 0.001).

Absolute cell counts were performed for stem/progenitor cells that were negative for markers of mature hematopoietic cells (T-cells, CD3; B-cells, B220; RBC, Ter-119; Myeloid cells, GR-1/Mac-1) while being positive for c-kit and Sca-1 (KSL, Figure 2(a)). Sickle mice had significantly fewer stem cells than control mice. We further quantified the number of a defined hematopoietic stem cell population (HSC; KSL/CD150+/CD48−) [27]. These cells were also significantly reduced in homozygous mice compared to controls (*t*-test, Figure 2(b)). As there were less stem cells in the bone marrow, we investigated the cell cycle status of HSC as a reduction in the number of quiescent cells (G_0_ of the cell cycle) has been associated with mobilization of HSC in normal tissues, and HSCs are reported to be mobilized in sickle cell patients [28].  Figure 2(c) shows a reduced number of cycling KSL cells suggesting a reduced number of quiescent stem cells in the bone marrow. We then tested the functional capacity of KSL cells using the *in vitro* high proliferative potential (HPP) assay. Sickle-derived stem cells formed significantly fewer colonies compared to control (*P* < 0.001, Figure 2(d)).

### 4.2. Oxidant Damage to Bone Marrow-Derived Cells

Using flow cytometric analysis and DCF and DHPE dyes, we measured the intracellular content of ROS and lipid peroxidation, respectively, in KSL cells. H_2_DCFDA becomes deacetylated by intracellular esterases as it crosses the membrane and becomes brightly fluorescent once oxidized by ROS producing DCF [29]. DHPE loses its fluorescence upon reaction with peroxy radicals [29]. Sickle-derived KSL cells demonstrated significantly increased lipid peroxidation and ROS compared to those derived from normal and hemizygous mice (*P* < 0.05, Figures 2(e)–2(f)). Importantly, in all of the above assays, the hemizygous mice showed an intermediate phenotype that correlated to their hematological defect.

### 4.3. Engraftment in the Sickle Microenvironment

**Figure 3:**
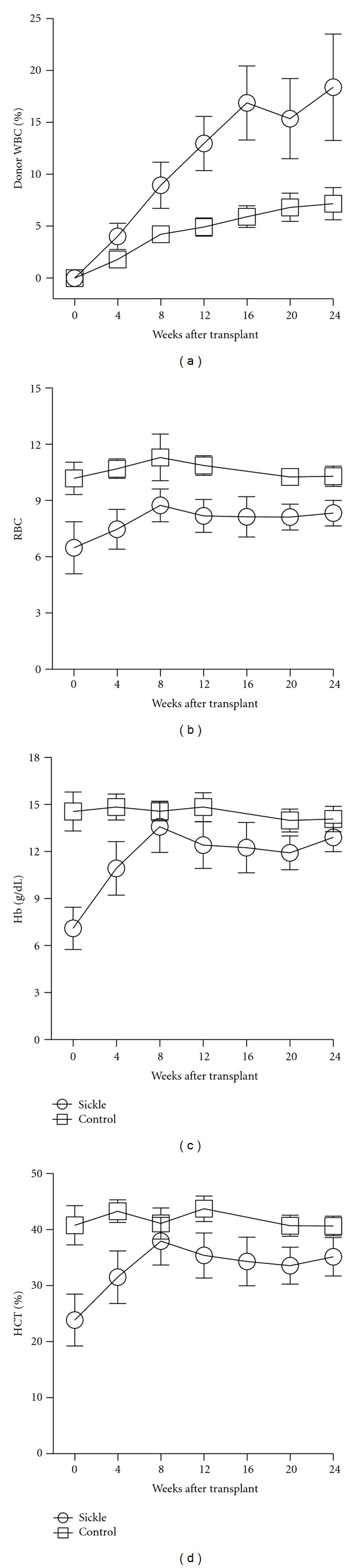
Preferential engraftment in sickle mice. (a) Peripheral WBC populations from normal mice engraft in homozygous sickle mice at a significantly (*P* < 0.01) faster rate than control mice when transplanted without ablation but in the presence of costimulation blockade. The levels of WBC engraftment coincide with (b)–(d) corresponding correction of RBC, hemoglobin, and hematocrit in recipient mice.

 We then addressed the issue of whether the sickle bone marrow environment was more conducive to engraftment by donor cells. To compare the engraftment efficiency of bone marrow cells into control (C57BL/6) and homozygous sickle mice (sickle), we transplanted 1 × 10^7^ control male bone marrow cells into mice receiving only costimulation blockade. As early as four weeks after transplantation, there was a significant increase in engrafted donor cells in the sickle mice compared to the control mice (*P* > 0.001, Figure 3(a)). This level of engraftment is remarkable considering the nonablative protocol, and the continued increase with time suggests that donor cells have a survival or proliferation advantage in the sickle environment. Peripheral RBC markers also showed correction of sickle hematology towards control levels indicating the survival advantage of normal over sickle RBC (Figures 3(b)–3(d)) [30].

### 4.4. Engraftment Capacity of Sickle Hematopoietic Cells

**Figure 4:**
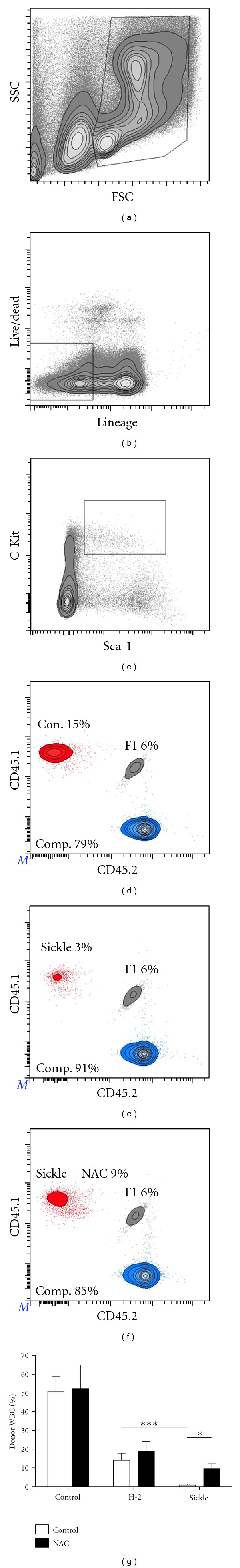
Functional defect of sickle hematopoietic stem cells in a competitive repopulation assay. Strategy for sorting KSL cells from whole bone marrow: mononuclear cells gated on FSC and SSC of whole BM (a), gated lineage and propidium iodide negative on mononuclear cells (b), and c-kit+, Sca1+, Lin− (KSL) cells (c). Peripheral blood engraftment after transplantation. Representative flow cytometry plots showing KSL cells from control mice engrafted at 15% (d), sickle KSL cells engrafted at 3% (e), and NAC- treated sickle KSL cells engrafted at 9% (f). Composite data showing a reduced capacity for engraftment between hemizygous and control mice and a further defect in KSL cells from homozygous mice 24 weeks after transplant (g). Graphical representation of the data from all mice: pretreatment of donor cells with NAC partially restored engraftment of KSL cells from homozygous sickle cell mice.

We then compared the engraftment potential of KSL cells derived from control, hemizygous, and homozygous sickle mice in a competitive repopulation assay to determine if sickle-derived KSL cells were functionally impaired. First, we bred male hemizygous sickle mice to female C57BL/6 mice to generate mice that expressed both CD45.1+ and CD45.2+ antigens on the surface of their cells (F1) allowing us to distinguish donor and host KSL as well as donor BM competitor cells. By HPLC, we confirmed that host F1 mice carried one copy of the human *β* sickle globin gene, as well as murine *α* and *β* globins (data not shown). For each experiment, KSL cells were derived from untreated, as well as NAC treated, control, hemizygous (H-2), and homozygous mice and transplanted into lethally irradiated F1 mice. Peripheral blood was analyzed at 4, 8, 12, 16, and 24 weeks after transplantation (Figure 4(a)). In two separate experiments, homozygous sickle-derived KSL cells demonstrated significantly reduced engraftment capabilities compared to control mice. NAC treatment improved engraftment of homozygous sickle KSL cells but did not fully correct the defect (Figure 4(b)). 

## 5. Discussion

Despite the benefits of hydroxyurea, people with SCD have limited treatment options with the only curative treatment remaining hematopoietic stem cell transplant (HSCT). While the numbers of children receiving transplant have continued to grow and now total in the hundreds, the majority are still performed with myeloablative conditioning (recently reviewed in [31]). The outcomes of these transplants in children are generally good with high levels of disease-free survival. However [32], there are significant concerns regarding long-term toxicities and complications from the preparative regimen, especially with regard to continued CNS complications and gonadal toxicities [33]. 

The continued desire to be able to offer transplant to a much larger selection of patients, including adults, has driven a number of trials utilizing a variety of nonmyeloablative protocols [34–36]. Most recipients have not had sustained engraftment, but Hsieh et al. successfully transplanted a small group of adults with mobilized peripheral blood from matched sibling donors [37]. These limited successes, continued issues with posttransplantation immunosuppression, and the limited number of HLA-matched donors will drive alternative transplant protocols and techniques for therapy with genetically corrected autologous cells.

These are similar findings to those in the murine model of Ataxia telangiectasia (ATM), which also demonstrated significant impairment of stem cell populations at least partially due to ROS and oxidative stress [38, 39]. Ataxia telangiectasia is a rare, recessive genetic disorder that causes neurological degeneration. The *Atm* gene controls DNA repair, cell cycle, and redox homeostasis. Ito et al. found that bone marrow-derived stem cells from *Atm* knock-out mice possess increased ROS and activated p38 MAPK that resulted in a reduction in KSL number, decreased colony-forming potential, loss of quiescence, and defective self-renewal capacity [38]. Similarly, sickle bone marrow has reduced colony-forming potential, and fewer sickle HSCs are in G_0_ suggesting that stem cells are mobilized. This is consistent with the high peripheral WBC count in both mice and humans and previous reports of mobilization in SCD. 

The oxidant damage of murine sickle HSC further translates into a functional defect in a reduced activity in a competitive repopulation assay. Interestingly, both hemizygote and homozygotes were affected. Antioxidant therapy in the form of NAC provided partial correction of the phenotype. Similar results were found in the ATM model where six weeks of treatment with either NAC or catalase antioxidants restored Atm^−/−^ HSC CFU ability to near normal levels [38].* In vivo* full HSC engraftment was only achieved when recipient mice were also treated with NAC. Oxidant-mediated HSC dysfunction is commonly seen in number of other model systems including Fanconi anemia (Fancc^−/−^) where ROS lead to increased apoptosis of Fancc^−/−^ cells [40]. A lack of FoxO family members also leads to an increase in ROS and a reduction in both HSC number and reconstitution ability [41]. In both of these models, antioxidant therapy with NAC is able to reverse or ameliorate the defects. Oxidant mediated stem cell damage is not limited to hematopoietic cells, for example, Kim and Wong demonstrated an oxidant-mediated defect in atm^−/−^ neural stem cells that was responsive to NAC [42]. It is interesting to speculate that the high levels of oxidant stress could affect other organ-specific stem cell populations, and that this might be an important factor in the ongoing repair of sickle-related organ pathology. The HSC defects should also be considered when designing gene therapy protocols first, as there may be a reduced number of HSCs available for collection and secondly as the HSCs have a reduced engraftment potential prior to *ex-vivo* manipulation.

Clinically, patients with severe sickle disease, who would be a desirable target population for HSCT, are likely to encounter more complications during transplant due to ongoing disease-related pathology and inflammation. However, with successful immunomodulatory strategies our data would suggest that engraftment of HSC into the sickle environment itself should be successful, and donor cells may have a comparative advantage. This could be especially important if designing approaches based on mixed hematopoietic chimerism that have been successfully used in murine model systems [30, 43].

All of the major pathologic consequences of SCD such RBC lysis, endothelial activation, and vaso-occlusion either induce or exacerbate the production of ROS with subsequent effects being likely to contribute to further pathologic processes (recently reviewed in [3, 4]). Consequently, a number of investigators have studied the use of antioxidants in SCD, mostly focusing on RBC effects. Vitamins C and E and NAC have all been shown to reduce ROS and increase the levels of glutathione in sickle RBC and PMN *in vitro* [21]. Treatment of NAC reduced the formation of dense RBC and increased the levels of intracellular glutathione in RBC of patients with SCD; importantly this correlated with a reduction in the number of vaso-occlusive crises during the treatment period [44]. Similarly, treatment with NAC for 6 weeks reduced phosphatidyl serine exposure on the membrane of sickle RBC and the levels of cell-free hemoglobin [45] indicating a cellular effect for an oral antioxidant.

In summary, we describe the effects of sickle-mediated oxidant stress on the bone marrow environment and hematopoietic stem and progenitors and detail defects in HSC function that raise important concerns when designing future stem cell therapies for sickle cell disease.
